# Validation of the Sudbury Vertigo Risk Score to risk stratify for a serious cause of vertigo

**DOI:** 10.1111/acem.70017

**Published:** 2025-03-11

**Authors:** Elliot Tissot van Patot, Danielle Roy, Elger Baraku, Kashyap Patel, Sarah McIsaac, Ravinder Singh, Daniel Lelli, Darren Tse, Peter Johns, Krishan Yadav, David W. Savage, Jeffrey J. Perry, Robert Ohle

**Affiliations:** ^1^ Medical Sciences Program, Faculties of Medicine and Science University of Dalhousie Halifax Nova Scotia Canada; ^2^ School of Epidemiology and Public Health, Faculty of Medicine University of Ottawa Ottawa Ontario Canada; ^3^ The Faculty of Medicine University of Ottawa Ottawa Ontario Canada; ^4^ Department of Critical Care, Department of Anesthesia Northern Ontario School of Medicine Sudbury Ontario Canada; ^5^ The Department of Neurology, Health Sciences North Health Sciences North Research Institute, Northern Ontario School of Medicine Sudbury Ontario Canada; ^6^ Department of Neurology and Otolaryngology University of Ottawa and Ottawa Hospital Research Institute Ottawa Ontario Canada; ^7^ Department of Emergency Medicine University of Ottawa and Ottawa Hospital Research Institute Ottawa Ontario Canada; ^8^ The Department of Emergency Medicine Northern Ontario School of Medicine Thunder Bay Ontario Canada; ^9^ The Department of Emergency Medicine Health Sciences North, Health Sciences North Research Institute, Northern Ontario School of Medicine Sudbury Ontario Canada

## Abstract

**Introduction:**

In 2022, nearly 0.5 million Canadians visited an emergency department (ED) for dizziness, accounting for over 3.5% of all ED visits. Of these patients, only 2%–5% received a serious diagnosis. The cost of ED and inpatient care for dizziness in Canada exceeds $200 million per year, of which neuroimaging accounts for a large proportion. Over one‐third of dizziness patients undergo a CT scan of the head, 96% of which are negative. Despite extensive investigation, patients discharged with a benign dizziness diagnosis have a 50‐fold increased risk of being admitted to the hospital within 7 days with a diagnosis of stroke. Our study aimed to derive a clinical risk score to guide the investigation and referral for serious causes of vertigo in ED patients.

**Methods:**

This multicenter historical cohort study was conducted over 7 years at three university‐affiliated tertiary care EDs. Patients presenting with vertigo, dizziness, or imbalance were recruited. The main outcome was an adjudicated serious diagnosis, defined as stroke, transient ischemic attack, vertebral artery dissection, or brain tumor. We estimated a sample size of 4450 patients, based on a 2% prevalence of serious outcomes, to evaluate the sensitivity with 95% confidence intervals (CIs).

**Results:**

A total of 4559 patients were enrolled (mean age 78.1 years, 57.8% women), with serious events occurring in 104 (2.3%) patients. The C‐statistic was 0.95 (95% CI 0.92–0.98). The risk of a serious diagnosis ranged from 0% for a score of <5 to 16.7% for a score >8. Sensitivity for a serious diagnosis was 100% (95% CI 96.5%–100%) and specificity was 69.2% (95% CI 67.8%–70.51%) for a score <5.

**Conclusion:**

The Sudbury Vertigo Risk Score effectively identifies the risk of a serious diagnosis in patients with dizziness. Thus, it guides further investigation, consultation, and treatment decisions and ultimately improves resource utilization and reduces missed diagnoses.

## INTRODUCTION

Vertigo is defined as a patient's illusion of motion, whether that be of themselves or their environment.^1^ It is a symptom common to various unrelated medical conditions and thus contributes to uncertainty in differential diagnoses.^2^ Matters are further complicated by the ambiguity of the term “vertigo.” The terms dizziness, unsteadiness, and vertigo are often used interchangeably by patients in the emergency department (ED).^3^ This paper will use the term dizziness to encompass vertigo, dizziness, and imbalance.

Accounting for more than 3.5% of ED visits in Canada in 2022, dizziness‐related ED care constitutes a significant proportion of healthcare costs (>$200 million in Canada and >$4 billion in the United States).^4^, ^5^, ^6^ Yet, only 2%–5% of patients presenting to the ED with a chief complaint of vertigo received a serious diagnosis. Patients presenting with a primary symptom of dizziness are often overinvestigated and underdiagnosed.^7^, ^8^, ^9^

There are currently no reliable clinical features that exclude serious causes of dizziness. This results in the overuse of neuroimaging and missed or delayed diagnoses of serious pathologies. Approximately 35% of patients presenting with dizziness are subjected to head computed tomographic (CT) scans, 98% of which show no abnormalities.^10^, ^11^ Despite the extensive investigation of dizzy patients, those discharged with a benign diagnosis are 50 times more likely to be admitted to the hospital within 7 days with a diagnosis of stroke compared to matched controls.^7^ Current efforts to stratify patient risk of a serious diagnosis have shown limited effectiveness (see discussion for full details).^12^, ^13^, ^14^, ^15^, ^16^ There have been two recent guidelines to help risk stratify patients with dizziness for a serious diagnosis. The American College of Emergency Physicians (ACEP) clinical policy recommends a standard comprehensive history and physical examination and the use of specific findings such as ABCD2 score, ocular motor examination, presence of additional neurologic deficits, and HINTS (if trained) to risk stratify patients with a possible stroke.^17^ The GRACE‐3 guidelines offer a more comprehensive approach using a symptom‐based approach assessing timing and triggers, focusing on the diagnosis of benign paroxysmal positional vertigo (BPPV) as the most common cause of vertigo in the ED and then using the HINTS in those with nystagmus and the STANDING algorithm as an alternative.^3^ Although there has been much discussion about how these guidelines differ they align in their recommendations that a full history and physical examination together with an assessment for vascular risk factors are important. The limitation of history and physical for posterior circulation stroke was highlighted in an accompanying systematic review to the GRACE‐3 guidelines.^18^ In addition, the HINTS examination is only applicable in those with acute vestibular syndrome a minority of dizzy patients. Lastly the STANDING algorithm, although a useful tool, has a sensitivity that is below the required threshold identified by ED physicians.^12^, ^13^, ^18^ Thus, developing a validated (external and temporal) clinical risk score to identify serious causes of vertigo in patients presenting with dizziness is essential to improve patient outcomes and care.

In response to the need for a validated approach, we developed the Sudbury Vertigo Risk Score (Table 1) to improve patient outcomes and care.^14^ This seven‐item tool can provide an accurate estimate of the probability that a patient with dizziness is suffering from a serious diagnosis (stroke, transient ischemic attack [TIA], vertebral artery dissection or brain tumor). Using a cut point of <5, the score has a sensitivity of 100% and a specificity of 72%.^14^ If validated, it could reduce unnecessary investigation in low‐risk patients and improve the efficiency and effectiveness of diagnosis and treatment of high‐risk patients. Our objective was to assess the accuracy of the Sudbury Vertigo Risk Score in a new cohort of patients presenting to the ED with dizziness.

**TABLE 1:** Sudbury Vertigo Risk Score.

Predictor	Points
Stroke risk factors	
Male	1
Age >65	1
Diabetes	1
Hypertension	3
Neurological deficits	
Motor/sensory	5
Cerebellar^a^	6
BPPV diagnosis	−5

Abbreviation: BPPV, benign paroxysmal positional vertigo.

^a^ Diplopia, dysarthria, dysphagia, dysmetria, ataxia.

## METHODS

### Study design

This retrospective multicenter cohort study was conducted in the EDs of three university‐affiliated urban Canadian tertiary care teaching hospitals from September 8, 2014, to June 10, 2020.

### Study population

We extracted data on consecutive alert patients 18 years and older, who presented to participating EDs with a chief complaint of acute vertigo, dizziness or imbalance and were assessed by an ED physician. Patients with symptom onset more than 14 days prior, head or neck trauma in the preceding 14 days, Glasgow Coma Scale score <15, systolic blood pressure <90 mm Hg, a syncopal episode in the preceding 14 days, or active cancer were excluded from the study. The research ethics board at each participating center approved the study without requiring written consent.

### Data collection

Five trained reviewers extracted data from multiple sources, including electronic ED records and consultant notes. All reviewers coded a subset of 20 charts to establish inter‐rater reliability. Kappa was calculated with the data extraction form considered a single variable, such that if any variable on the form varied between reviewers, it was counted as a disagreement.

Chart abstractors underwent training (didactic session and five charts joint review) and testing (10 charts dual independent review); when testing resulted in a kappa of >0.8 between the trainer and tester, they were validated for independent data abstraction. We followed guidelines for historical cohort studies put forward by Jansen et al.^15^

Data were entered into a computerized database using Statistical Analysis System (SAS) software. Data management and study coordination were conducted at the Health Sciences North Research Institute.

### Outcome measures

The primary outcome of a serious diagnosis was defined as a diagnosis of stroke, TIA, vertebral artery dissection, or brain tumor diagnosed in the ED or within 30 days of the initial assessment. Outcomes were defined as follows: Stroke (ischemic and hemorrhagic)—rapidly developed clinical symptom(s) of focal (or occasionally global) disturbance of cerebral function lasting more than 24 h or until death with no apparent nonvascular cause; TIA—sudden, focal neurological deficit lasting for <24 h, presumed to be of vascular origin, and confined to an area of the brain or eye perfused by a specific artery; brain tumor—radiological evidence of an intracranial mass that another more likely diagnosis cannot explain that required intervention (medical or surgical) within 30 days of diagnosis; and vertebral artery dissection—radiological evidence of vertebral artery dissection, hematoma, or pseudoaneurysm.

#### Outcome assessment

The primary outcome was assessed for all patients from a composite of sources, including site hospital records and autopsy reports at the site hospital. An adjudication committee, blinded to the initial ED visit, reviewed all possible outcome events. The adjudication committee comprised three members: a stroke neurologist and two experienced emergency physicians. These assessors independently evaluated each possible outcome, and an event was considered to have occurred if at least two of the three physicians agreed. Secondary outcomes followed a similar process.

### Data analysis

Descriptive statistics were computed using frequencies and proportions for categorical variables and means and standard deviations for continuous variables. We compared proportions and mean differences using the chi‐square test or Fisher exact test as appropriate and *t*‐test, respectively. A two‐sided *p*‐value below 0.05 suggested a statistically significant difference.

The optimism and optimism‐correct C‐statistic were calculated. We assessed the calibration of the model and score using a calibration slope between observed and predicted probabilities at each score category. Because of the small number of patients and events in the higher risk scores, we collapsed the scores above 14. Where more than one variable data was missing from a patient, they were excluded from the analysis. We did not assess for nonlinearity.

We assessed the impact of the score on resource utilization using the score level that would define a low‐risk group with 0 serious diagnoses. To provide the most conservative estimate, we assumed no CT would be performed in the low‐risk group, but every patient in the medium‐ and high‐risk groups would now undergo a CT.

All statistical analyses were performed using SAS software Version 9.4. Based on the method proposed by Riley et al.,^16^ we estimated a required sample size of 85 outcome events. This was based on the assumption of a shrinkage of 0.9, Cox‐Snell R squared of 0.1, an outcome proportion rate between 0.02 and 0.05, and a model based on up to 20 predictors. We adhered to TRIPOD reporting guidelines.^19^

## RESULTS

**FIGURE 1:**
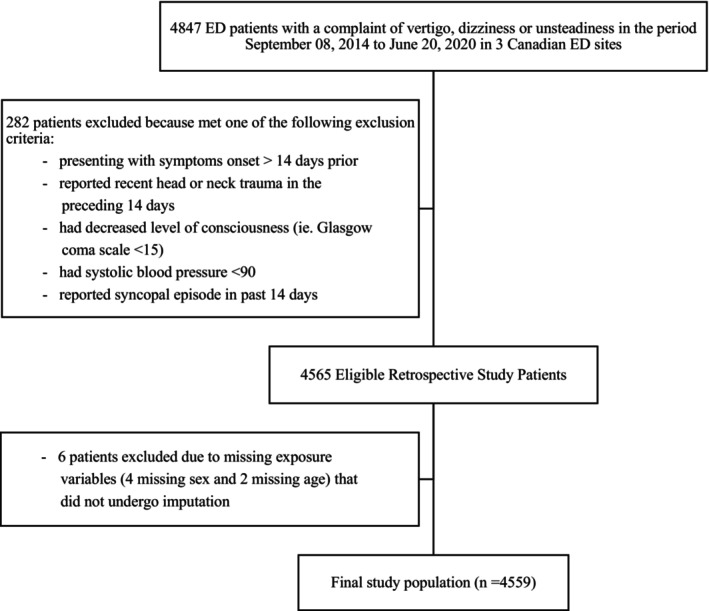
Flow diagram of the study cohort.

We included 4559 (mean age, 78.1 years; 57.8% female) patients presenting with vertigo, dizziness or imbalance between September 08, 2014, and June 10, 2020. Figure 1 displays the breakdown of exclusions resulting in the final study cohort.

Within the study population, 1678 (36.8%) CT scans, 349 (7.7%) CT angiographies, and 253 (5.5%) magnetic resonance imaging scans were performed. A total of 104 (2.3%) patients received a serious diagnosis, of whom 91 (2.0%) were diagnosed with a stroke, six (0.1%) with a TIA, five (0.1%) with a brain tumor, and two (0.04%) with a vertebral artery dissection (Table A1).

The baseline characteristics of patients in the study cohort are presented in Table 2. Age over 65, previous stroke, TIA, hypertension and diabetes, dysphagia, diplopia, motor deficit, sensory deficit, ataxia, dysarthria, dysmetria, headache, hearing loss, vomiting, nystagmus, dizziness with a gradual onset, dizziness lasting more than 2 min, single episode, and persistent dizziness when still were associated with receiving a serious diagnosis. Clinical features significantly less associated with a serious cause included dizziness triggered by a change in any position as well as a clinical diagnosis of BPPV (Table A1).

**TABLE 2:** Baseline characteristics of patients presenting to the ED with dizziness according to stroke events.

Predictors	Serious outcome (*n* = 4559)	
	Yes (*n* = 104)	No (*n* = 4455)
Sex, female	38 (36.54)	2600 (58.36)
Age 65 or over	66 (63.46)	1854 (41.62)
Heart rate (beats/min)^a^	77.10 (±15.30)	77.74 (±15.99)
Systolic blood pressure (mm Hg)	153.6 (±28.3)	141.4 (±22.8)
Diastolic blood pressure (mm Hg)	85.2 (±15.1)	81.3 (±12.5)
Past medical history		
Previous stroke	20 (19.23)	170 (3.82)
Previous TIA	7 (6.73)	113 (2.54)
Hypertension	88 (84.62)	2785 (62.51)
Diabetes	27 (25.96)	643 (14.43)
Atrial fibrillation	7 (6.73)	152 (3.41)
Neurological deficits		
Dysphagia	4 (3.85)	18 (0.40)
Diplopia	15 (14.42)	100 (2.24)
Motor deficit	32 (30.77)	61 (1.37)
Sensory deficit	16 (15.38)	38 (0.85)
Ataxia	67 (64.42)	589 (13.22)
Dysarthria	22 (21.15)	51 (1.14)
Dysmetria	13 (12.50)	45 (1.01)
Symptoms		
Nausea	48 (46.15)	1876 (42.11)
Vomiting	35 (33.65)	887 (19.91)
Headache	35 (33.65)	1088 (24.42)
Neck pain or discomfort	8 (7.69)	159 (3.57)
Facial eye pain	2 (1.92)	47 (1.05)
Hearing loss	7 (6.73)	109 (2.45)
Tinnitus	7 (6.73)	277 (6.22)
Recent viral URTI symptoms	2 (1.92)	237 (5.32)
Timing		
Ongoing	77 (74.04)	3040 (68.24)
Gradual	19 (18.27)	514 (11.54)
Abrupt	64 (61.54)	2466 (55.35)
More than 2 min	55 (52.88)	1570 (35.24)
Episodes		
Single	39 (37.50)	977 (21.93)
Multiple	37 (35.58)	2110 (47.36)
Movement triggers		
Head turning	6 (5.77)	657 (14.75)
Getting up	10 (9.62)	345 (7.74)
Lying down	2 (1.92)	139 (3.12)
Bending over	1 (0.96)	74 (1.66)
Looking up	0	41 (0.92)
Rolling over in bed	0	69 (1.55)
Walking	2 (1.92)	76 (1.71)
Any	27 (25.96)	1363 (30.59)
Persistent when still	29 (27.88)	646 (14.50)
Physical examination		
Unable to walk unaided	46 (44.23)	550 (12.35)
Can walk more than 10 steps	46 (44.23)	3746 (84.09)
Nystagmus	13 (12.50)	176 (3.95)
BPPV diagnosis	1 (0.96)	614 (13.78)

*Note*: Data are provided as *n* (%) or mean (±SD).

Abbreviations: BPPV, benign paroxysmal positional vertigo; TIA, transient ischemic attack; URTI, upper respiratory tract infection.

^a^ Missing observations for heart rate (*n* = 31 + 2325), systolic blood pressure (*n* = 30 + 2325), and diastolic blood pressure (*n* = 30 + 2329).

For a score <5, the risk of a serious outcome was 0%, with a sensitivity for a serious diagnosis of 100% (95% confidence intervals [CI] 96.5%–100%) and a specificity of 69.2% (95% CI 67.8%–70.51%). The risk of a serious diagnosis in patients with a score of 5 to 8 was 0.9%. In those with a score >8, the risk of a serious diagnosis increased to 16.7% (Table A2). Using a threshold of >7, the risk score had a sensitivity for a positive finding on CT of 100% (95% CI 94%–100%) and a specificity of 84.2% (95% CI 83.1%–85.2%; Table A3).

**FIGURE A1:**
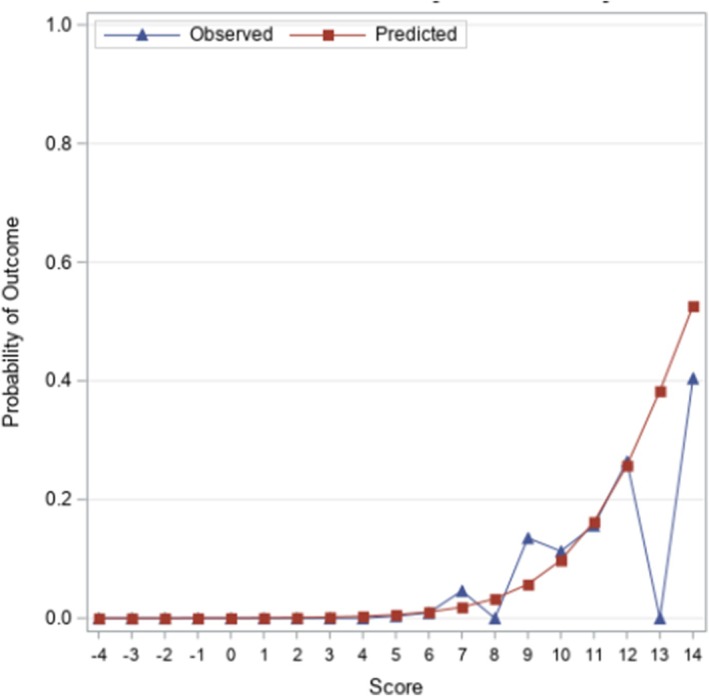
Observed and predicted probability of a serious outcome by risk score.

**FIGURE A2:**
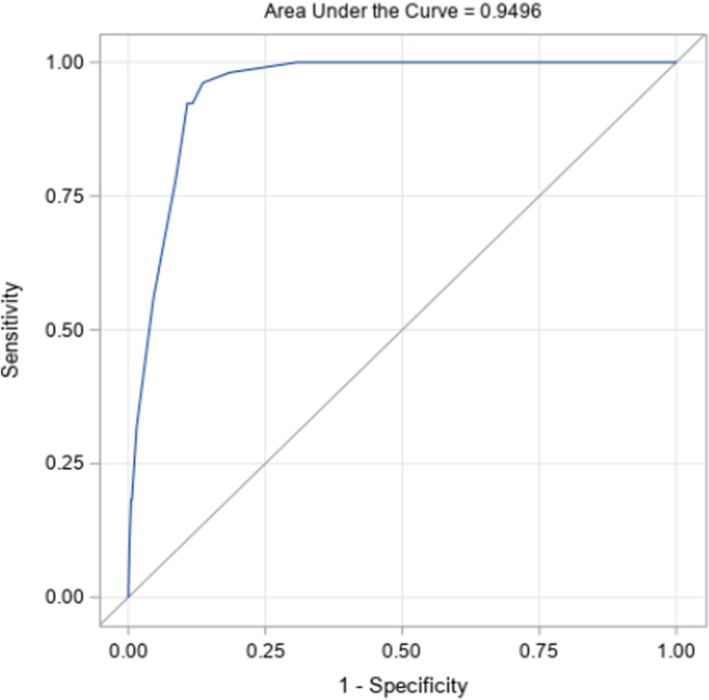
Receiver operating characteristic (roc) curve for observed probability of outcome by risk score.

The calibration plot for the validation cohort shows a slope of 1.0 for the observed versus the expected risk for a serious diagnosis, and the model calibration line is very close to the ideal calibration line (Figure A1). The area under the curve was 0.95 (95% CI 0.92–0.98; Figure A2).

## DISCUSSION

This retrospective multicenter cohort study validated the accuracy of the previously derived Sudbury Vertigo Risk Score.^20^ Using a threshold score of <5, the risk score can accurately identify over two‐thirds of the patient cohort as low risk (Table A1). These patients do not require further investigation or treatment for a serious diagnosis of vertigo. Notably, nearly half (45%) of all advanced imaging was observed within this low‐risk group (Table A1). The risk score can estimate the probability of a serious outcome, which ranges from 0% for a score of <5% to 40.43% for a score above 13 (Table A3). By identifying those at low risk who can be safely discharged without further investigation and triaging patients at a higher risk for a serious outcome, this risk score can improve patient outcomes and care, minimize missed or delayed diagnoses, and reduce unnecessary costs to the health care system.

### Previous studies

In two surveys, ED physicians reported a need for a clinical risk score to help assess dizziness patients. They defined a required miss rate of <1%.^21^, ^22^ Five clinical decision aids/scores have been derived, all subject to small sample size and a high risk of bias. Chen et al. retrospectively derived a clinical risk score from a 1:1 case control cohort from patients admitted to a neurology ward. Cases were those discharged with a diagnosis of posterior circulation stroke or TIA; controls were a random selection of patients admitted during the same period. They identified nine risk factors: high blood pressure, diabetes, ischemic stroke, rotating and rocking, difficulty in speech, tinnitus, limb and sensory deficit, gait ataxia, and limb ataxia. The score had a sensitivity of 94.1% (95% CI 89.1%–97.3%) and a specificity of 41.4% (95% CI 33.5%–49.7%). This is likely an optimistic estimate as case control studies will falsely elevate estimates of sensitivity and specificity.^23^ This study had a stroke incidence of 50% (>10 times the expected number), indicating significant selection bias.^24^

Kuroda et al.^25^ conducted a retrospective study of 498 patients, with a stroke incidence of 29.4%. They used multivariate logistic regression to derive the *TriAGe + score* consisting of eight variables: triggers, atrial fibrillation, male, hypertension, brainstem or cerebellar dysfunction, focal weakness, speech impairment, and dizziness and no history of dizziness. This score had a sensitivity of 77.5% and a specificity of 72.1% not sufficiently accurate to rule out a serious cause for a patients dizziness.

Bi et al. performed a prospective multicenter cohort study, with two neurologists recruiting 790 from 2360 eligible patients, with a stroke prevalence of 10%. They derived a *nomogram for stroke risk assessment* based on sex, trigger, isolated symptoms, nausea, history of brief dizziness, high blood pressure, finger nose test, and tandem gait assessment. The model demonstrated a high diagnostic accuracy (sensitivity 100%, 95% CI 95.49%–100.00%) and specificity of 57.75% (95% CI 54.02%–61.41%) but has not been validated. Clinical decision aids derived based on neurology‐assessed clinical variables have failed prospective validation, with significantly lower diagnostic accuracy when performed by ED physicians.^12^, ^13^, ^26^ The derivation cohort had twice the expected stroke prevalence; this could indicate spectrum bias that would artificially inflate sensitivity estimates.^23^ The nomogram requires use of a computer, limiting its usability.^27^

Yamada et al.,^28^ through assessment of a case series of posterior circulation strokes, decided on a three‐item checklist called the *Defensive Stroke Scale*: sensory disturbance, ataxia, or ocular deficit. If any of these are present, a stroke cannot be ruled out. They retrospectively assessed 1014 patients who presented to the ED with dizziness and found a sensitivity of 100% (95% CI 96.4%–100%). They excluded 65 due to poor documentation and had a stroke prevalence of 10.5%. There was no consistent application of an outcome assessment, with 16% of patients having stroke ruled out by ED physician clinical assessment. The spectrum bias, retrospective nature of the study, and potential for missed outcomes increase the probability that the tool is unlikely to maintain its 100% sensitivity on external validation.

The *HINTS examination* is composed of three assessments (head impulse test, nystagmus, and test of skew). The HINTS examination applies only to patients with acute vestibular syndrome, which make up approximately 10%–20% of those presenting with vertigo.^13^, ^29^ The HINTs examination has been validated for use by neurologists maintaining a high diagnostic accuracy; however, there is conflicting evidence of the accuracy when performed by ED physicians. Dmitriew et al.^13^ found a specificity of 64.3% in an AVS population and 96.4% in a mixed population, though no central causes were identified, precluding sensitivity calculation. Gerlier et al.^12^ demonstrated that after a focused training program (4 h of lectures and 2 h of workshops by otology experts), ED providers achieved a high sensitivity (97.9%) for stroke detection but moderate specificity (64.5%) in a mixed population. When pooled with studies where the performer was unspecified, the combined sensitivity for stroke identification was 83.4% and specificity 88.9%. Inter‐rater reliability studies, however, revealed mixed results: poor agreement (0.29–0.40) in Kerber et al.^8^ potentially influenced by differences in examiners' training (emergency medicine vs. neurology). These findings suggest that while HINTS can be highly sensitive for identifying central causes, its performance depends on examiner expertise and consistent training. Studies evaluating the HINTS+ (Head Impulse, Nystagmus, Test of Skew with added hearing assessment) battery in patients with acute vestibular syndrome (AVS) report a sensitivity of 97.1% (95% CI 71.2%–99.8%) and a specificity of 85.6% (95% CI 66.2%–94.8%) for detecting central causes, such as stroke. These results are derived from four studies where the HINTS+ examination was conducted exclusively by neurologists. Notably, no studies have evaluated the performance of ED providers using the HINTS+ battery in AVS populations, emphasizing the need for research into how well ED physicians can apply this diagnostic tool and whether targeted training could bridge this gap in practice.^18^

Vanni et al.^20^ developed the *STANDING algorithm*, which consists of the (1) discrimination between spontaneous and positional nystagmus, (2) evaluation of the nystagmus direction, (3) head impulse test, and (4) evaluation of equilibrium, the first and second of which are components of the HINTS examination. A 2022 systematic review of diagnostic accuracy studies of this algorithm reported sensitivity for identifying a serious cause of dizziness from 93.4% to 100% and specificity from 71.8% to 94.3%.^20^, ^21^ The review found all three studies of this algorithm were at a high risk of bias. The only external validation study found a sensitivity of 93.6% (95% CI 84.3–98.2) and a specificity of 74.8% (95% CI 84.3–98.2), with all physicians rating confidence in assessing the nystagmus and head impulse test as low.^12^, ^21^

Comparatively, the Sudbury Vertigo Risk Score has a sensitivity of 100% (95% CI 96.5%–100%) and a specificity of 69.2% (95% CI 67.8%–70.5%). This fulfills accuracy requirements identified by emergency physicians, achieving a posttest probability of <0.5% for a serious diagnosis.^22^

Two guidelines offer advice on the risk stratification of patients presenting with dizziness for a serious outcome. The ACEP clinical policy, although not specifically directed at patients with dizziness, does offer advice for this patient population. It promotes the use of history and physical examination assessment to help risk stratify patients and, if trained, the use of the HINTS examination.^17^ The GRACE‐3 guidelines endorse the approach that includes the use of the HINTS examination, STANDING algorithm and assessment of BPPV.^3^ As often is the case of clinical guidelines neither approach as diagnostic algorithms have been externally validated for accuracy or impact on clinical practice. That being said both guidelines offer a reasonable a practical approach to the risk stratification of patients with dizziness.

## STRENGTHS

This retrospective cohort study was conducted over three EDs across Canada, including both rural and urban academic sites, thus improving external validity. Temporal validation was achieved by including an entirely new population of patients in the study cohort. To address the spectrum bias often seen in derivation studies, the study cohort included a representative sample of ED patients with a chief complaint of vertigo, as demonstrated by the rates of serious outcomes. Patients enrolled in the study received a diagnosis from ED physicians, which contributes to high level of generalizability. Implementation blinded adjudication committees to assess serious outcomes ensured a rigorous event classification.

## LIMITATIONS

As a retrospective analysis, the variables were not standardized, potentially introducing variability in the data. Additionally, no new sites were included beyond those in the derivation study, which may limit the generalizability of the findings. The outcome assessment was incomplete, as not all patients underwent a criterion standard assessment, potentially leading to misclassification of outcomes. This could artificially increase the sensitivity with outcomes at risk of being classified as nonoutcomes if they do not undergo the appropriate diagnostic test. This has the potential to increase the sensitivity of the risk score artificially; however, in the prospective derivation phase, no new strokes were identified during telephone follow‐up. The calculation of the risk score was centralized at the coordinating site, and thus the score's accuracy may have been impacted by the clinician's ability to compute it correctly. Therefore, the actual diagnostic accuracy may differ in clinical practice. Furthermore, the use of the WHO definitions for stroke and TIA may overestimate the true number of ischemic strokes by including stroke mimics. These limitations highlight the need for caution in interpreting the results and for a prospective multicenter validation study of the Sudbury Vertigo Risk Score in diverse clinical settings. One of the components of the score is a clinical diagnosis of BPPV; this includes a positive Dix–Hallpike or supine roll or a discharge diagnosis of BPPV. As this was a retrospective review, we could not assess inter‐rater reliability of this or any other components. The accuracy of diagnosis of BPPV could vary between physicians. This could affect the sensitivity and specificity of the score if validated at a different center.

### Clinical implications

This study highlights the potential utility of the Sudbury Vertigo Risk Score in predicting serious outcomes in patients presenting to the ED with dizziness. Nearly half of all CTs were in the low‐risk group. In addition, no serious outcomes were identified on CT in a score <8. There is the potential of using different cut points to guide different investigations, treatments, or referrals. If this score is prospectively validated in centers not included in the derivation cohort, the next step will be a consensus meeting. This meeting will need to include neurologists, radiologists, ED physicians, ENT surgeons, and patients. The goal would be to establish the most appropriate investigations and treatments at each serious outcome probability level.

## CONCLUSIONS

The Sudbury Vertigo Risk Score presents a promising tool to provide guidance in clinical decision making for ED physicians. Provided it is prospectively validated. By utilizing variables available at the bedside, that clinicians have indicated a high degree of comfort in assessment, the risk score is both effective and practical. The Sudbury Vertigo Risk Score has significant potential to enhance clinical decision making, optimize the usage of health care resources, and improve patient outcomes in the management of vertigo.

## FUNDING INFORMATION

This project was supported through grants from the Northern Ontario Academic Medical Association and Physician Services Incorporated Foundation.

## CONFLICT OF INTEREST STATEMENT

The authors declare no conflicts of interest.

## Data Availability

The data that support the findings of this study are available on request from the corresponding author. The data are not publicly available due to privacy or ethical restrictions.

## APPENDIX A

**TABLE A1:** Univariate and multivariate logistic regression analysis of serious cause predictors in patients presenting to the emergency department with dizziness (*n* = 4559).

Predictors	Crude OR	Adjusted OR
Sex (%), (ref = female)	2.43 (1.63–3.65)	2.83 (1.74–4.63)
Age		
Below 65	Reference	Reference
Over 65	2.44 (1.63–3.65)	2.00 (1.20–3.34)
Past medical history		
Previous stroke	6.00 (3.60–10.01)	
Previous TIA	2.77 (1.26–6.11)	–
Hypertension^a^	3.30 (1.93–5.64)	2.25 (1.18–4.29)
Diabetes	2.08 (1.33–3.25)	0.91 (0.52–1.60)
Atrial fibrillation	2.04 (0.93–4.48)	–
Neurological deficits		
Dysphagia	9.87 (3.28–29.68)	0.92 (0.16–5.39)
Diplopia	7.34 (4.10–13.13)	4.79 (2.31–9.91)
Motor deficit	32.02 (19.67–52.11)	10.32 (5.40–19.73)
Sensory deficit	21.14 (11.36–39.33)	15.57 (6.48–37.41)
Ataxia	11.89 (7.88–17.92)	7.81 (4.82–12.65)
Dysarthria	23.17 (13.43–39.98)	13.94 (6.42–30.29)
Dysmetria	14.00 (7.30–26.85)	2.70 (1.10–6.62)
Symptoms		
Nausea	1.18 (0.80–1.74)	–
Vomiting	2.04 (1.35–3.09)	–
Headache	1.57 (1.04–2.37)	–
Neck pain or discomfort	2.25 (1.08–4.71)	–
Facial eye pain	1.84 (0.44–7.67)	–
Hearing loss	2.88 (1.31–6.34)	–
Tinnitus	1.09 (0.50–2.37)	–
Recent viral URTI symptoms	0.30 (0.09 = 1.42)	–
Physical exam		
Unable to walk unaided	5.63 (3.79–8.38)	–
Can walk more than 10 steps	0.15 (0.10–0.22)	–
Nystagmus	3.47 (1.91–6.33)	–
Timing		
Ongoing	1.33 (0.85–2.07)	–
Gradual	1.71 (1.03–2.84)	–
Abrupt	1.29 (0.87–1.92)	–
More than 2 mins	2.06 (1.40–3.05)	–
Episodes		
Single	2.14 (1.43–3.20)	–
Multiple	0.61 (0.41–0.92)	–
Movement triggers		
Head turning	0.35 (0.16–0.81)	–
Getting up	1.27 (0.66–2.46)	–
Lying down	0.61 (0.15–2.49)	–
Bending over	0.58 (0.08–4.18)	–
Looking up	0.73 (0.00–3.26)	–
Rolling over in bed	0.43 (0.00–1.90)	–
Walking	1.13 (0.27–4.66)	–
Any	0.80 (0.51–1.24)	–
Persistent when still	2.28 (1.47–3.53)	–
BPPV diagnosis	0.06 (0.01–0.44)	0.09 (0.01–0.67)

^a^ Reference categories are defined as no; hypertension defined as history of or prevalent hypertension at presentation.

**TABLE A2:** Score to predict risk of central outcome in patients presenting to the ED with dizziness.

Risk score	Observed probability of stroke (%)
−4	0
−3	0
−2	0
−1	0
0	0
1	0
2	0
3	0
4	0
5	0.36
6	0.90
7	4.60
8	0
9	13.51
10	11.27
11	15.63
12	26.42
13	0
14	0
15	49
16	50
17	57.14

**TABLE A3:** Central outcome for each risk score by diagnostic test in derivation cohort.

Risk score	*n*	All events	Stroke	Tumor	TIA	MS	Medication use in patients with events/total			Event outcome by diagnostic modality		
							Plavix	ASA	DAPT	Outcome positive on CT/total performed	Outcome positive on CTA/total performed	Outcome positive on MRI/total performed
Total	4559	104	91	5	6	2	8/53	20/320	2/24	62	25	44
−4	93	0	0	0	0	0	0	0/1	0	0/7	0	0
−3	87	0	0	0	0	0	0/1	0/5	0/1	0/13	0/2	0/1
−2	24	0	0	0	0	0	0	0/1	0	0/3	0/1	0/2
−1	79	0	0	0	0	0	0	0/3	0	0/11	0	0/2
0	761	0	0	0	0	0	0/2	0/20	0/1	0/174	0/20	0/13
1	561	0	0	0	0	0	0/4	0/24	0/2	0/148	0/18	0/10
2	148	0	0	0	0	0	0	0/11	0	0/45	0/9	0/3
3	434	0	0	0	0	0	0/3	0/8	0/1	0/132	0/11	0/12
4	894	0	0	0	0	0	0/5	0/62	0/3	0/327	0/48	0/17
5	551	2	1	0	1	0	0/8	0/60	0/4	0/226	0/31	1/28
6	221	2	2	0	0	0	0/6	0/27	0/1	0/97	0/26	1/18
7	87	4	4	0	0	0	0/2	0/8	0/1	2/54	0/16	0/9
8	43	0	0	0	0	0	0	0/8	0	0/25	0/10	0/5
9	111	15	13	2	0	0	1/1	3/10	1/1	8/74	5/20	6/20
10	204	23	20	0	2	1	2/5	3/21	1/2	14/141	5/46	12/45
11	160	25	22	1	2	0	2/7	7/31	0/4	14/117	4/42	11/38
12	53	14	11	1	1	1	2/5	3/11	0/1	8/43	4/20	7/16
13	1	0	0	0	0	0	0	0	0	0/1	0/1	0
14	6	0	0	0	0	0	0	0	0	0/4	0/2	0/3
15	20	8	8	0	0	0	0/1	1/5	0/1	7/18	3/11	3/6
16	14	7	6	1	0	0	0/2	3/4	0/1	5/12	2/10	2/4
17	7	4	4	0	0	0	1/1	0	0	4/6	2/5	1/1
